# Nuclear clustering—manifestations of non-uniformity in nuclei

**DOI:** 10.1098/rsta.2023.0123

**Published:** 2024-07-23

**Authors:** T. Uesaka, N. Itagaki

**Affiliations:** ^1^ RIKEN Nishina Center for Accelerator-Based Science, Saitama 351-0198, Japan; ^2^ RIKEN Cluster for Pioneering Research, Saitama 351-0198, Japan; ^3^ Graduate School of Science and Engineering, Saitama University, Saitama 338-8570, Japan; ^4^ Department of Physics, Osaka Metropolitan University, Osaka 558-8585, Japan; ^5^ Nambu Yoichiro Institute of Theoretical and Experimental Physics (NITEP), Osaka Metropolitan University, Osaka 558-8585, Japan

**Keywords:** nuclear cluster, origin of elements, alpha decay, tensor interaction, spin–orbit interaction, knockout reaction

## Abstract

Well-developed α clusters are known to exist in light N=Z nuclei, and their properties are reasonably well described with modern nuclear structure theories. However, ‘modestly’ developed clusters in medium to heavy nuclei remain little understood, both theoretically and experimentally. Extension of the focus to include modestly developed clusters leads us to a concept of ‘generalized clusters’ and ‘cluster ubiquitousness’. The former includes clusters more weakly bound than an α cluster, such as deuteron, triton and He3 , and even clusters partially broken owing to nuclear medium effects. The latter means the existence of clusters in any nuclei, where cluster development was not previously discussed. Effects of the tensor and the spin–orbit interactions on the coexistence of clusters with independent nucleons are discussed using recent nuclear theoretical models. A mixture of the clusters with shell-like components plays an essential role in the synthesis of elements in the universe and the origin of life, together with an α decay. It is also pointed out that clustering in heavy nuclei may accelerate fission and fusion processes. Future experimental plans using cluster knockout reactions, which have the potential to extract information of ‘generalized clusters’ in a variety of nuclei including stable and unstable nuclei, are also discussed.

This article is part of the theme issue ‘The liminal position of Nuclear Physics: from hadrons to neutron stars’.

## 1. To be uniform or not to be uniform

It is no exaggeration to say nuclear physics started with a manifestation of non-uniformity, i.e. clustering in nuclei. The α decay, where an α cluster formed in nuclei is spontaneously emitted from a heavy nucleus, was among the first radioactive decays discovered by Becquerel in 1896. It was identified as the emission of a He4 nucleus by Rutherford in 1898 [1]. Energetic α particles emitted in α decays were used to discover a nucleus as a tiny and heavy core of an atom through elastic scatterings [2] and to elementally transform nitrogen into oxygen through the N14⁢(α,n)⁢O17 reaction [3].

The α decay was modelled by Gamow as quantum-mechanical tunneling of an α particle formed in a nucleus through the Coulomb barrier [4]. The model successfully explained the α -decay lifetime spreading over many orders. Observations of α decays and the success of Gamow’s theory let people believe that a nucleus is made of α particles, and nuclear models with α particles as fundamental constituents, together with protons and electrons, had been developed. One of the first α cluster models by Wheeler [5] was proposed in 1937 against this background.

The situation changed from the mid-1930s to the late 1940s; in analogy to atomic shell structure, ideas to consider that a nucleus forms shells of protons and neutrons sprouted just after the discovery of neutrons [6,7]. The shell picture is based on the independent motions of protons and neutrons, and the resulting nucleon distribution is uniform. This is contrary to the non-uniform picture of the α cluster models, where two protons and two neutrons are spatially correlated. This shell picture was later completed by Mayer & Jensen through the introduction of strong spin–orbit interaction [8,9], which results in the energy splitting between the spin doublet comparable with a major shell gap. This strong spin–orbit interaction is one of the features characteristic of nuclear systems. In parallel, a liquid-drop model based on a strong coupling picture, seemingly completely opposite to that of the shell picture, was proposed by Bohr [10]. The model also presumes uniformity of a nucleon distribution. In [10], it is said ‘It is even very doubtful that α particles exist in nuclei in the manner assumed in present theories of α -ray decay.’

In spite of the general success of shell and liquid-drop theories without *a priori* assumption of the explicit non-uniformity in nuclei, the occurrence of α clusters manifests the significance of non-uniformity in some cases. One of the most famous examples is the $0^{+}$ ground state of Be8 , which is located just above (0.091 MeV) the threshold to decay into two α particles.

**Figure 1.**
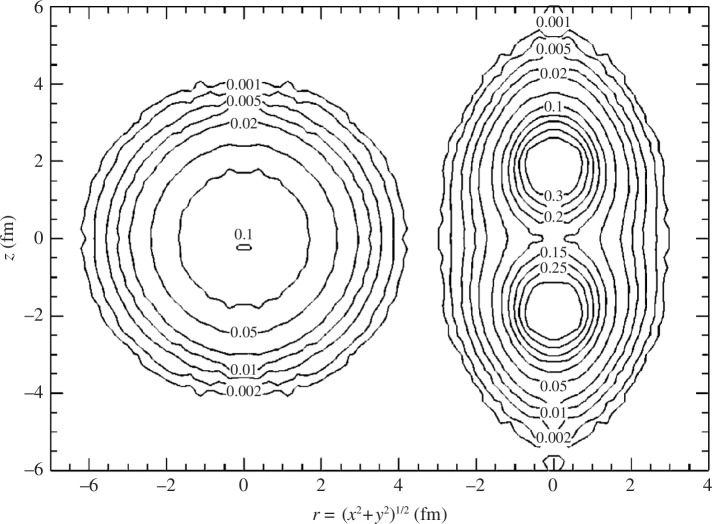
Constant-density contours for Be8 in the laboratory (left) and the intrinsic (right) frames calculated with the quantum Monte Carlo theory. Reprinted from [11]. ©2000 with permission from the American Physical Society.

As shown in figure 1, the appearance of a two- α structure has been shown by the modern *ab initio* calculation [11]. The state has a finite but unusually long half-life of $6.7\pm 1.7\times 10^{-17}$ s since the two He4 nuclei have to experience the long tunneling through the Coulomb barrier before the decay. From the ground state, a rotational band structure is formed, and the first $2^{+}$ state (excitation energy of $E_{x}=3.030$ MeV) and the first $4^{+}$ state ( $E_{x}=11.35$ MeV) fit into the energy interval typical for the rotation of a rigid rotor. The nucleus has no other excited states up to the $E_{x}=15$ MeV region suggesting that a simple α – α picture holds quite well.

Another famous example of the cluster structure is the second $0^{+}$ state of C12 called the Hoyle state. The state at $E_{x}=7.654$ MeV is located just above the threshold energy ( $E_{x}=7.275$ MeV) to decay into three α clusters. The state is considered to have played an essential role in the formation of carbon from three α s in stars, as is discussed in §3a. Many microscopic calculations have shown that the state has a well-developed cluster structure [12,13].

The other example that indicates the necessity of clustering is the first excited state of O16 . The spin-parity of the first excited state at $E_{x}=$ 6.049 MeV is observed to be $0^{+}$ , the same as the ground state. The large excitation energy serves as the signature for the doubly closed shell ( p -shell closure) structure of the ground state at the proton and neutron numbers of 8. However, the spin-parity of the first excited state contradicts a simple-minded prediction of the shell model, because a one-particle-one-hole excitation to the s⁢d -shell from the closed p -shell should result in a negative parity state. The first excited state in O16 had been therefore called ‘the mysterious $0^{+}$ state’, and there have been serious debates in the 1960s. Eventually, this mystery was solved by interpreting this state with a C12+α cluster configuration.

Now, the existence of well-developed clusters in light nuclei, in particular near the α decay threshold in the N=Z nuclei, is widely accepted and reasonably well described with modern nuclear structure theories [14]. However, their coexistence with shell-like (independent nucleon) components remains to be fully understood. As will be discussed later, the mixing of shell-like components is intrinsic to the synthesis of elements essential to our society and life. The mixing should be more pronounced in the ground and excited states of medium to heavy nuclei, where ‘modestly’ developed clustering occurs, which leads to an α decay and can accelerate nuclear fission and fusion processes. Obviously, understanding how clusters coexist with independent nucleons, namely, in a symbiotic (mutually beneficial) or competitive (mutually exclusive) way, is quite an important and fascinating topic in current and future nuclear physics. The degree of mixing reflects the nature of the nuclear interactions, in particular, the balance between the two non-central interactions, the tensor and the spin–orbit interactions.

Extension of focus to include modestly developed clusters leads us to a concept of ‘generalized clusters’ and ‘cluster ubiquitousness’. The former includes clusters more weakly bound than an α particle, such as deuteron, triton and He3 , and furthermore, clusters partially broken owing to nuclear medium effects. The latter means the existence of clusters in any nuclei, even in nuclei where the cluster development was not previously discussed.

The present article outlines the physics behind the relationship between cluster and shell degrees of freedom, and nuclear phenomena induced by the mixture of both degrees of freedom in light to heavy nuclei. Experimental perspectives using cluster knockout reactions, which have the potential to extract information on ‘generalized clusters’ in a wide range of nuclei including stable and unstable nuclei, are also discussed.

## 2. Theoretical background of cluster formation and its dissolution

### (a) Tensor interaction promoting the cluster structure

As mentioned above, well-developed α clusters are found in light nuclei, in particular N=Z ones, as depicted in the famous Ikeda diagram [15]. The concept of strong binding inside clusters and weak interaction for the relative motion between them is the guiding principle for the emergence of cluster structures; clusters must be strongly bound to maintain the structure as subsystems, and the relative interaction must be weak enough to keep the inter-cluster distance. What can be the origin of these features? The answer is the tensor interaction, one of the non-central nucleon–nucleon interactions mainly owing to the pion exchange mechanism.

It has been known that the contribution of the tensor interaction is quite significant in the formation of He4 . The benchmark *ab initio* calculation for He4 using the AV8’ interaction has shown that the contribution of the tensor interaction in the potential energy of He4 is -68 MeV [16], more than that of the central interaction ( -55 MeV). Therefore, the tensor interaction strengthens the binding of He4 and makes it a good subsystem.

Not only that, but the stabilization mechanism owing to the tensor interaction serves as a trigger for the increase of the inter-cluster distances. The tensor correlation in a cluster induces the two-particle-two-hole excitation, where two interacting nucleons are excited to higher energy orbits. This two-particle-two-hole excitation is partially Pauli-blocked in the nuclear medium. Therefore, clusters tend to keep the relative distances to prevent the Pauli-blocking effect [17].

### (b) Spin–orbit interaction promoting the shell structure

The strong spin–orbit interaction inevitable for the magic numbers 28, 50, 82 and 126 [8,9] causes an interesting competition between the clustering feature and the shell structure. According to the shell model, each nucleon performs single-particle motion in a one-body field with a good total angular momentum j in addition to the orbital angular momentum ℓ and the spin s . On the one hand, the spin–orbit interaction consolidates this symmetry and drives the formation of the shell structure. On the other hand, this spin–orbit interaction destroys the cluster structure by weakening the spatial correlation of the proton–proton (or neutron–neutron) pair with opposite spin orientations. This is owing to a character in the spin–orbit interaction that boosts spin-up nucleons and spin-down nucleons in opposite directions.

### (c) Quasi clusters—partially dissolved clusters

Owing to the contribution of the spin–orbit interaction, which becomes stronger with increasing mass number, the clusters are partially dissolved in the low-lying states of the nuclear systems. Such partially broken clusters owing to the spin–orbit effect can be regarded as ‘quasi clusters’. The quasi-cluster structure contains both features of shell and cluster structures. Interplay between the tensor and the spin–orbit interactions determines properties of the quasi cluster and thus the mixing or symbiosis of cluster and shell structures. This is important in many cases, including the synthesis of C12 , as will be discussed in §2d. The antisymmetrized quasi-cluster model (AQCM) [18] is a theoretical model that enables the description of the transition from the cluster structure to the shell structure owing to the spin–orbit interaction by introducing a single parameter in the wave function, and the quasi-cluster structure is an intermediate state of this transition. By extending the definition of the cluster structure to quasi-cluster, we can expect various clustering features in a wide range of mass number.

### (d) Modern *ab initio* calculations and clustering

The appearance of the cluster structure in light nuclei has been demonstrated by modern *ab initio* calculations, where realistic nucleon–nucleon interactions have been adopted. The quantum Monte Carlo calculation has successfully shown the formation of two α cluster structures in Be8 [11] (see figure 1). The unitary correlation operator method combined with the Fermionic molecular dynamics has also shown the appearance of a three- α cluster configuration in the Hoyle state and a mixture of the shell-model-like components in the ground state [19].

**Figure 2.**
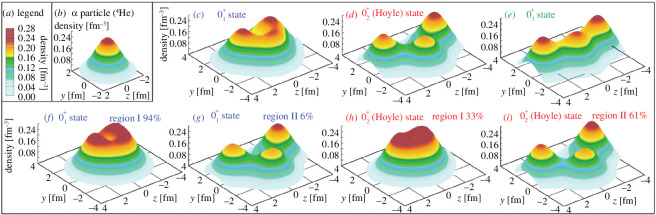
Density profiles of α and C12 nuclei obtained with the *ab initio* Monte Carlo shell model. (*a*) Colour code of the density. (*b*) Density of the α -particle ground state. (*c*–*e*) Density of three $0^{+}$ states of C12 . (*f*–*i*) Decomposition into the region I (‘liquid drop’) and II (‘clustering’). Reprinted from [20]. ©https://creativecommons.org/.

The recent Monte Carlo shell model calculation shown in figure 2 has confirmed that the second $0^{+}$ state is a three- α state, where various three- α configurations mix [20]. Also, the ground state ( $0_{1}^{+}$ ) is a mixture of shell-model-like and cluster-model-like configurations; the α clusters are melting but we can identify the remnant there, which is consistent with the quasi-cluster point of view. There has been great progress in the chiral effective field theory and Monte Carlo lattice calculations, and low-lying states of C12 , including the Hoyle state, have been described [21]. Also, the cluster structure of C12 is investigated by the no core shell-model approach [22].

Challenges to describe cluster structure with various modern *ab initio* approaches will be promising in light nuclei. However, its application to medium to heavy nuclei, in particular open-shell nuclei, would be very difficult even with future supercomputers.

### (e) Possible clustering in medium and heavy nuclei

Clustering is discussed even in medium to heavy nuclei, especially in nuclei with a doubly magic core + 4 nucleons, e.g. Ti44,52 , Te104 and Po212 , although it has been naively expected that the spin–orbit effect increasing with the mass number and the weakening of the tensor effect in the saturated density would make clustering less favourable. For instance, the appearance of both the positive-parity and negative-parity bands is predicted and observed in ${\;}^{44}Ti$ [23], suggesting the existence of an asymmetric cluster structure such as ^40^Ca + ^4^He.

**Figure 3.**
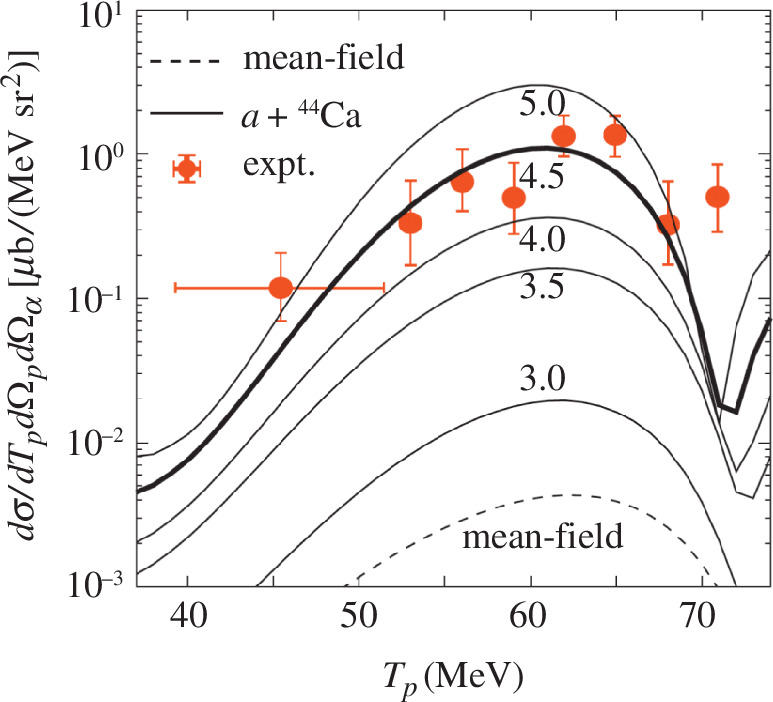
Triple-differential cross-section of the ${\;}^{48}Ti(p,p\alpha {)}^{44}Ca$ reaction. Experimental data from [24]. are compared with DWIA calculations. Reprinted from [26]. ©2021 with permission from the American Physical Society.

Recently, the reanalysis of the Ti48⁢(p,p⁢α)⁢Ca44 data taken at $E_{p}=$ 100 MeV [24] raises a debate. From Ti48 , which is thought to have less α cluster structure compared with Ti44 and Ti52 [25], a certain α -knockout cross-section is observed as shown with red circles in figure 3. However, the theory based on the distorted-wave impulse approximation (DWIA) using the Ti48 wave function obtained with antisymmetrized molecular dynamics (AMD) without an explicit assumption of α cluster structure (indicated as ‘mean field’) gives two orders of magnitude smaller cross-section than the experimental data [26]. The small theoretical cross-section is probably owing to the fact that the α cluster structure is significantly washed out by the strong spin–orbit contribution in the AMD calculation. It should be noted that the Gogny interaction employed in the AMD calculation does not include a tensor interaction term. This example illustrates the current situation of our understanding on the clustering in medium to heavy nuclei. For better understanding, the introduction of the tensor interaction effects [27], which are missing in many theoretical calculations at present, together with the low-density effects discussed in §5, will be a promising step.

## 3. Clustering in light nuclei: origins of elements and life

### (a) Synthesis of carbon and clustering

Some cluster states are inevitable for the existence of elements heavier than carbon and thus of life. He4 is the second abundant element in the universe and continues to be produced in stars through the p⁢p chain^1^ , where He4 is produced from four protons. However, since there is no stable nucleus with the mass numbers A=5 and 8, the path of simple proton captures is closed.

**Figure 4.**
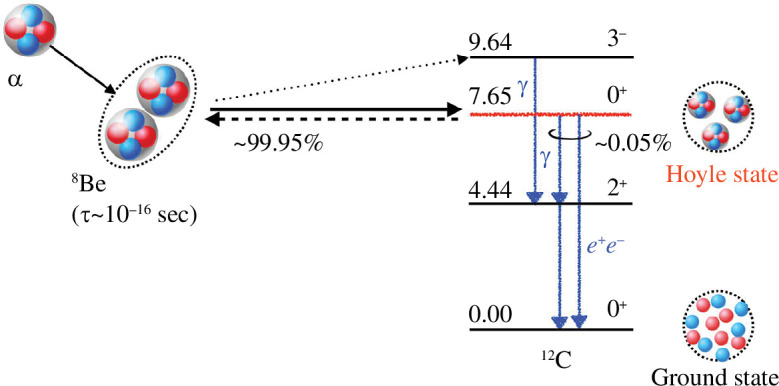
Schematic figure for the formation of C12 : The triple- α reaction through a formation of the Be8 resonance state populates the Hoyle state at 7.654 MeV. The Hoyle state decays to three α s (~99.95%) and the C12 ground state via electromagnetic transitions (~0.05%).

This serious problem of the prevention of nucleosynthesis beyond A=5 and 8 was solved by Salpeter & Hoyle; Salpeter suggested that C12 is produced via the triple- α reaction [28], and Hoyle predicted that there must be a resonance state just above the three- α threshold to account for the abundance of helium, carbon and oxygen [29]. The ‘unusually’ long lifetime of the Be8 nucleus, discussed in §1, makes it possible for the third α to participate in the synthesis. Soon after the prediction, the resonance state was observed as the second $0^{+}$ state of C12 , and now known as the Hoyle state. Similarly, the C12⁢(α,γ)⁢O16 reaction is crucial to explain the abundance ratio of carbon and oxygen. These two examples demonstrate the significance of nuclear clustering for the origin of elements and life. Figure 4 depicts the triple- α reaction and the de-excitation of the Hoyle state.

Theoretically, such well-developed α cluster states as the Hoyle state can be expressed as the mixing of clusters in different spatial arrangements. Already in the 1970s, the terminology of a ‘gas-like state’ has been used in describing the Hoyle state with well-developed three- α clusters. The α clusters are weakly interacting just like gas, although their internal binding is strong. More recently, these gas-like cluster states have been reinterpreted as α boson states. The authors of [30] have proposed the Tohsaki–Horiuchi–Schuck–Röpke (THSR) wave function, which is capable of describing gas-like α cluster states in a transparent way. Here, all the α clusters are equally treated as bosons, but the wave function is fully antisymmetrized and the Pauli exclusion principle is taken into account at the nucleon level. The size of the system is governed by a single parameter, and a single THSR wave function has been found to quantitatively describe the well-developed cluster states and have a large overlap with the solutions of the microscopic α cluster models. Besides reproducing the well-developed cluster states of ${\;}^{8}Be$ and C12 , the THSR wave function has predicted the existence of gas-like states of four- α clusters ( O16 ) and five- α clusters ( Ne20 ) [31]. Recently, the candidates for the gas-like α -cluster states of O16 and Ne20 were experimentally observed slightly above the threshold energies to decay into four α s and five α s, respectively [32]. In Be8 and C12 , the gas-like cluster states appear just above the corresponding threshold energies, but with increasing the number of the clusters, the energies of the gas-like states slightly shift higher owing to the Coulomb repulsion among the α clusters as suggested in [33].

### (b) Remaining questions about the Hoyle state and beyond

The key quantities for the synthesis of three α s to C12 via the Hoyle state are the γ -decay ( E⁢2 transition) width of the second $0^{+}$ state going to the first $2^{+}$ state ( $E_{x}=4.440$ MeV), which is a bound state, and its ratio to the α -decay width [34]. Although many microscopic three- α cluster models succeeded in reproducing the properties of the Hoyle state and the elastic and inelastic form factors of the low-lying states of C12 , they underestimated the B⁢(E⁢2) value for this transition by less than half. This has been a crucial problem of the microscopic cluster model calculations. This is owing to the fact that the γ -decay width is strongly affected by shell-like components mixed with the α cluster components.

In addition, it turned out that if both the ground state and the Hoyle state have pure three- α cluster structures, the Hoyle state becomes too spatially extended owing to the orthogonality to the ground state. Furthermore, the intrinsic configuration of the Hoyle state and the $2^{+}$ state, which is a rotational state of the ground state, become too different. The mixing of α -breaking components in the ground and $2^{+}$ states was found to overcome this disagreement; the second $0^{+}$ state slightly shrinks by the mixing of α -breaking components in the ground state and the transition to the $2^{+}$ state drastically increases [19,35]. This is a good example to demonstrate the role played by the interplay between the cluster and shell structures [36].

Recently, it has been reported from the experimental side that this E⁢2 transition probability from the second $0^{+}$ to the first $2^{+}$ state of ^12^C might be much larger than the previously accepted value of $\Gamma_{\mathrm{rad}}=3.81\,\left(0.39\right)\times 10^{-3}$ eV. The ^12^C $\left({p,p}^{\prime }\right)$ reaction experiment with proton- γ - γ triple coincidence measurement has shown that the radiative width of the Hoyle state is determined to be $\Gamma_{\mathrm{rad}}=5.1\,\left(6\right)\times 10^{-3}$ eV [37]. This value is about 34% larger than the adopted value and is considered to impact models of stellar evolution and nucleosynthesis.

At the normal stellar temperature of $T_{9}∼0.1$ ( $T_{9}=10^{9}$ K), the triple- α reaction process is mainly caused via the Hoyle state. However, with increasing temperature, other higher resonances could contribute to this reaction, especially the first $3^{-}$ state at $E_{x}=9.642$ MeV, which is a well-known state with a three- α cluster structure. This state is the first negative-parity state of C12 , and the symmetry of equilateral-triangular shape ( $D_{3\,h}$ ) allows the formation of rotational band structures starting with $0^{+}$ and $3^{-}$ , and thus the $3^{-}$ state is considered to be the counterpart of the $0^{+}$ ground state. The state mainly α -decays, but recently, the E⁢1 electric transition from the $3_{1}^{-}$ state to the $2_{1}^{+}$ state at $E_{x}=4.440$ MeV has been measured; the radiative-decay probability has been observed to be $1.3_{-1.1}^{+1.2}\times 10^{-6}$ [38].

The E⁢1 transition is the lowest order electromagnetic transition of the multipole expansion; however, this transition is forbidden between isospin zero states. In light N=Z nuclei including α and C12 , protons and neutrons have essentially the same wave functions and the isospin of the system becomes zero. The initial state of this transition, the $3^{-}$ state, consists dominantly of three- α clusters and has isospin zero. The final state, the $2^{+}$ state also has isospin zero. Consequently, the E⁢1 transition is caused by the tiny mixing components of the isospin symmetry breaking, which is induced by the symmetry-breaking terms of the Hamiltonian such as the Coulomb interaction. The result suggests that the $3_{1}^{-}$ state noticeably enhances the triple- α reaction rate. Although it had been considered that the three- α reaction rate at $T_{9}>2$ is significantly smaller than the estimation in the NACRE compilation [39], the new rate comes back to that in NACRE within its uncertainty.

### (c) Synthesis of oxygen to magnesium

^16^O is the most abundant nucleus of oxygen, one of the most important elements for the existence of life. The abundance, in turn, brings about the formation of all the heavier elements. ^16^O is formed by the ${\;}^{12}C+\alpha \to {\;}^{16}O+\gamma$ reaction [40], where there is no resonance state within the Gamow window (typical energy range of $E_{x}=300$ keV). Thus, the synthesis from the C12+α continuum state is considered to be dominant, contrary to the formation of ${\;}^{12}C$ from the three α s via the prominent resonance (Hoyle) state. Here, the $1^{-}$ and $2^{+}$ subthreshold states in ${\;}^{16}O$ also add important contributions to the low-energy capture cross-section. There have been extensive investigations into the ${\;}^{12}C+\alpha$ reaction rate, but significant uncertainty remains. Experimentally, the problem comes from poor accessibility to the low-energy region owing to the Coulomb barrier.

In the formation of ${\;}^{16}O$ from ${\;}^{12}C$ and ${\;}^{4}He$ , again the violation of the isospin symmetry is important. The E⁢1 transition owing to tiny isospin-breaking components in the ${\;}^{12}C+\alpha$ continuum contributes comparably with the E⁢2 transition. The theoretical predictions require a precise description of the ${\;}^{12}C+\alpha$ continuum including the isospin breaking of the α and ${\;}^{12}C$ clusters. This is another interesting subject where a partial breaking of clusters leads to nucleosynthesis, but remains to be a challenging theoretical problem.

The cluster-shell interplay also contributes synthesis of heavier elements. In Mg24 , various cluster configurations such as C12 + C12 and O16 + α + α have been discussed. In the 1960s, the ${\;}^{12}C$ + C12 reaction became experimentally feasible, and one of the first pieces of evidence observed was the presence of sharp resonant states around the Coulomb barrier top energy [41]. The states have been called molecular resonance states, where the C12 + C12 cluster configuration keeps a ‘molecular shape’ for some amount of time. With the development of the accelerators and the increase in bombarding energy, the observation of the molecular resonances has shifted to much higher energy regions. In the 1990s, there was much debate on the possible existence of a six- α linear-chain configuration in high excitation energy region of Mg24 [42]. Also, the research of molecular resonance has been extended to other nuclei, such as the C12 + O16 and O16 + O16 cluster configurations in Si28 and S32 , respectively. Recently, C12 + C12 fusion reaction at an energy much below the Coulomb barrier top has been attracting attention in connection with explosive phenomena in the universe, such as carbon burning in massive stars and X-ray superbursts [43]. The resonances within the Gamow window compel fusion reactions in the astrophysical environment.

## 4. Dynamical production of clusters in heavy nuclei beyond lead

### (a) α preformation probability—the century-old question

Clustering determines the stability and dynamics of heavy nuclei beyond lead.

An α decay is among the most well-known nuclear phenomena and is used in a variety of applications: americium–beryllium ( Am241-Be ) sealed neutron sources, detection of smoke in a fire alarm box and cancer therapy using At211 and Ac225 [44].

Since Gamow has explained the α decay as quantum mechanical tunneling of α through the Coulomb barrier, nuclear physicists have continued efforts to predict the α preformation factor using nuclear structure models such as shell models, cluster models and their hybrids. Although the shell model is among the most successful models and has explained many nuclear properties, the model considerably underestimates the α preformation factors [45].

On the other hand, the cluster models that presume the existence of α clusters *a priori* predict more reasonable α preformation factors in particular for Po212 , which can be well described as a two-body system of a doubly magic Pb208 nucleus and an α cluster. However, the extension of the model to other nuclei is not so trivial. Recently, the THSR approach discussed in §3a has been applied to α -decay nuclei [46].

**Figure 5.**
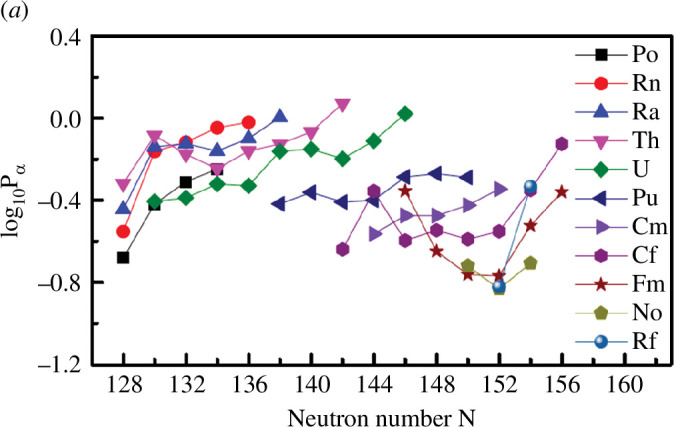
α preformation factors in the model presented in [47] versus the neutron number of parent nuclei for the decays of even–even isotopes with Z=84-104 . Reprinted from [47]. ©2018 with permission from the American Physical Society.

Figure 5 shows the α preformation factors in even–even isotopes with Z=84−104 , extracted from the α -decay lifetime using the prescription in [47]. They are known to be in the range of 0.01–1 [47,48], which means only a $10^{-5}$ to $10^{-3}$ fraction of all nucleons forms the α cluster.

The quantitative description of the α preformation factor is a homework problem of the last century and remains a big challenge. One of the clues in solving this problem is the development of α clusters around the low-density surface of a nucleus. This point will be revisited in §5.

### (b) Acceleration of nuclear fission by α cluster formation

Nuclear fission, a factor that determines the lifetimes of heavy elements along with an α decay, can also be a playground of clusters.

**Figure 6.**
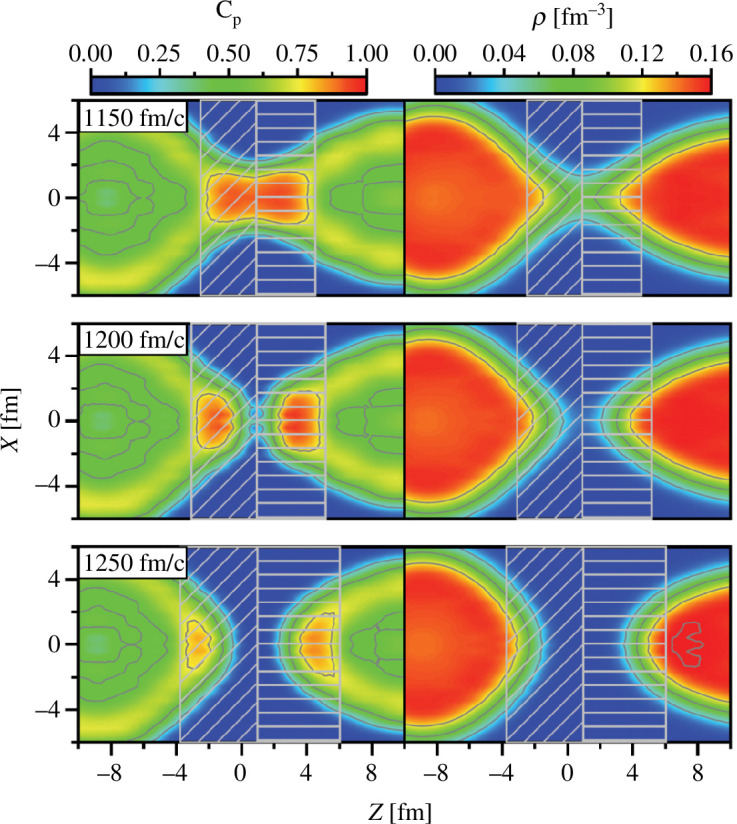
The proton localization functions $C_{p}$ (left) and a nucleon density ρ (right) at t= 1150, 1200 and 1250 fm/c in the fission of Pu240 . Reprinted from [49]. ©2022 with permission from the American Physical Society.

Recent time-dependent density functional theory predicts that the formation of α clusters accelerates the fission of heavy nuclei [49]. Figure 6 shows calculated values of the localization function [50] for the protons $C_{p}$ (left) and the nucleon density ρ (right) in the fission of Pu240 . A unity localization function $C_{p}∼1$ corresponds to equal densities for four types of nucleons (proton spin-up, proton spin-down, neutron spin-up and neutron spin-down), namely the occurrence of an α cluster. The top, middle and bottom panels show immediately before ( t= 1150 fm/c), at the moment of (1200 fm/c) and immediately after (1250 fm/c) the scission. The calculated localization function clearly exhibits the appearance of α clusters in the low-density neck region. The analysis also clarifies that the Coulomb repulsion between the α clusters accelerates scission to two fragments.

### (c) Can α cluster formation help nuclear physicists to produce superheavy elements?

In 2015, it was declared that the seventh period of the periodic table was completed with discoveries of Z= 113 (nihonium), 115 (moscovium), 117 (tennesine) and 118 (oganesson). Immediately, nuclear physicists began a challenge to pioneer the eighth period starting with the 119th element. Cross-sections of the cold and hot fusion reactions with emission of one or a few neutrons, respectively, used for the production of superheavy elements so far, are becoming much smaller as Z increases.

Instead, fusion reactions accompanying α emission together with a few neutrons, the so-called αyn reaction [51], may lead to a breakthrough in the production of elements in the eighth period. Actually, αyn -reaction cross-sections are known to exceed those for hot fusion reactions in the neutron-deficient actinide region. Apparent advantages of α emission over neutrons are fast removal of large internal energy and angular momentum. The formation of an α cluster in the low-density regions discussed above may reinforce the strengths of the αyn reactions over fusion reactions without α emission. Future experimental efforts with large-acceptance separators and theoretical studies with cluster degrees of freedom will quantify how promising the αyn reactions will be. This may open better access to the eighth period.

## 5. Experimental challenges in search of ‘generalized clusters’ and ‘cluster ubiquitousness’

### (a) Clustering in medium-mass nuclei

In the previous sections, we have seen that nuclear clusters play vital roles in light nuclei with Z≤20 and in heavy nuclei beyond lead ( Z=82 ). Are there non-negligible cluster components in nuclei between the two regions? How ubiquitous are clusters in the nuclear chart?

Some nuclear reaction data strongly indicate the necessity of clustering in nuclei [52]. Multi-nucleon transfer reactions such as (p,α) , (d,Li6) and α knockout reactions are good examples.

**Figure 7.**
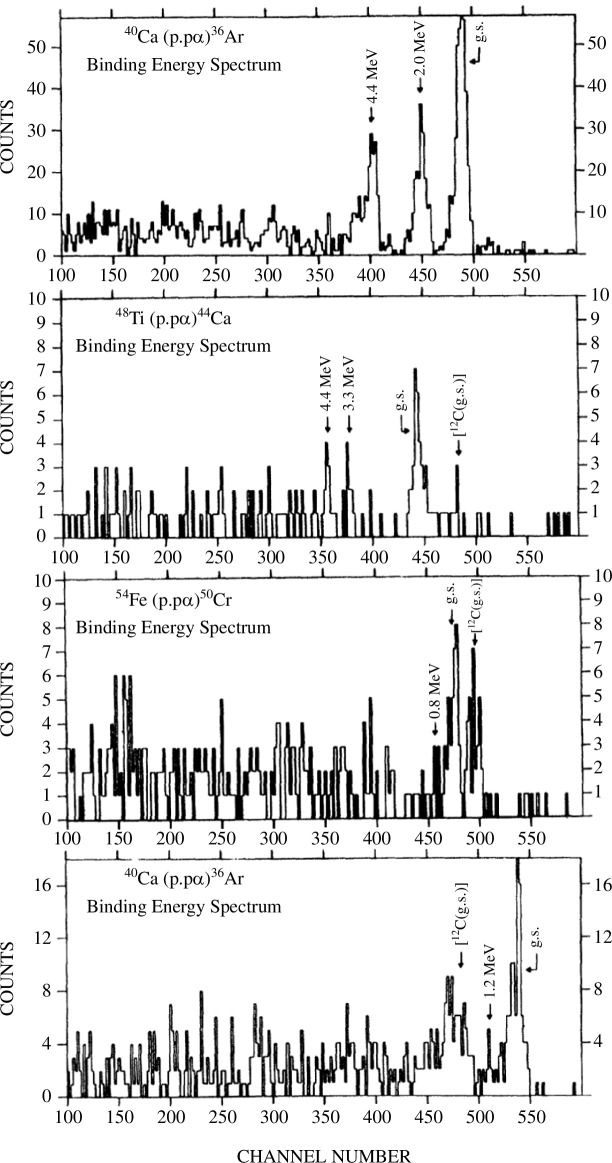
Separation energy spectra of the (p,p⁢α) reactions at $E_{p}=100$ MeV for Ca40 , Ti48 , Fe54 and Zn66 . Reprinted from [24]. ©1984 with permission from the American Physical Society.

Figure 7 shows separation energy spectra of the (p,p⁢α) reactions at $E_{p}=100$ MeV for Ca40 , Ti48 , Fe54 and Zn66 [24]. In [24], results for O16 , Mg24 , Ne20 , Sinat and S36 are also reported. In all cases, peaks corresponding to the removal of an α particle from the medium mass nuclei are clearly observed. The (p,p⁢α) data for Ne20 and Ti48 are reanalyzed using DWIA [26,53]. The DWIA calculations with α -core wave functions calculated with AMD are found to reproduce the Ne20⁢(p,p⁢α) data quite reasonably [53] but fail to describe the Ti48 data as discussed in §2e (see figure 3). This fact reminds us that our understanding of cluster structure is limited to the case of well-developed ones ( ${\;}^{20}Ne={\;}^{16}O+\alpha$ ) and essential physics about ‘modestly’ developed clusters and their coexistence with shell-like components remains to be understood. The interplay between the tensor and spin–orbit interactions discussed in §2 can be one of the keys.

In the discussed pieces of evidence, clusters are found to be persistent against the effect of the spin–orbit interaction, which becomes stronger as the number of nucleons increases.

Note that the heavy-ion collision is another way to discuss the formation of clusters in the nuclear medium [54].

### (b) α knockout reaction studies of stable Sn isotopes

A recent experiment [55] motivated by predictions of the relativistic mean field theory [56] has shown an interesting feature of α clustering at the nuclear surface. In [56], the formation of α clusters at the surface of Sn isotopes is discussed based on the generalized relativistic density functional theory with the inclusion of explicit cluster degrees of freedom. The theory predicts that α particles appear in the dilute region at the surface of heavy nuclei, and the formation probability depends on the growth of the neutron skin. Furthermore, one of the most important consequences is that the α formation probability monotonically decreases with the number of neutrons in Sn isotopes.

The α cluster knockout experiment on Sn112-124 at the RCNP cyclotron facility has exhibited the neutron number dependence of the α formation probability [55]. In the experiment, a scattered proton and a knocked-out α particle from the Sn112-124⁢(p,p⁢α) reaction induced by a 392 MeV proton were analyzed with the double-arm spectrometer consisting of the Grand RAIDEN and the LAS.

**Figure 8.**
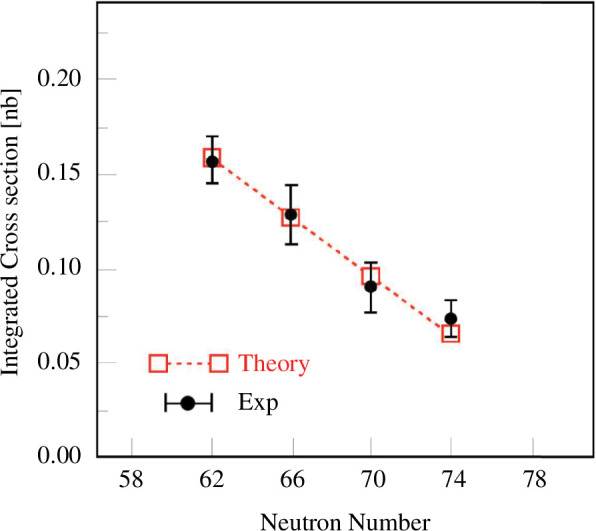
The neutron-number dependence of the Sn112-124⁢(p,p⁢α) cross-section integrated over transitions to low-lying states of the residual Cd isotopes. Data are taken from [55].

Figure 8 shows the neutron-number dependence of the cross-section integrated over the low-energy peak [55]. The integrated cross-section apparently decreases with the neutron number, which is fairly consistent with the prediction in [56]. It should be noted that a similar conclusion was also drawn from the (d,Li6) data in [57]. The agreement between theory and experiments hints that α clusters develop at the dilute surface of nuclei, and its formation probability is significantly affected by a neutron–proton asymmetry. The former is closely related to an assumption in the α preformation theories discussed in §4a.

The above experimental and theoretical studies indicate:

— It is reasonable to expect that clustering develops at the dilute surface region of *any* nuclei.
— Neutron number (isospin) dependence can be a key to understanding cluster formation mechanisms.
— (Proton-induced) knockout reactions are useful experimental tools to extract information on clustering in nuclei.

### (c) Experimental perspectives on clustering in medium to heavy nuclei using knockout reactions

As we have seen above, mechanisms of clustering in nuclei are relevant to a variety of aspects of the structure and stabilities of both finite nuclei and infinite nuclear matter but remain yet to be fully understood. The clues provided by the Sn112-124⁢(p,p⁢α) results may lead to a revelation of unknown aspects of clustering in nuclei and their relevance to other aspects of nuclear structure.

Recently, the ONOKORO project started aiming at the comprehensive investigation of clustering in nuclei in the mass region of A=36-222 using cluster knockout reactions. The project extends the previous works in [24,55] in three directions: (i) all of the light clusters, deuteron, triton, He3 and α , are investigated. (ii) A longer isotopic chain is covered using stable and unstable nuclei. (iii) A wider mass region up to A=222 is covered.

**Figure 9.**
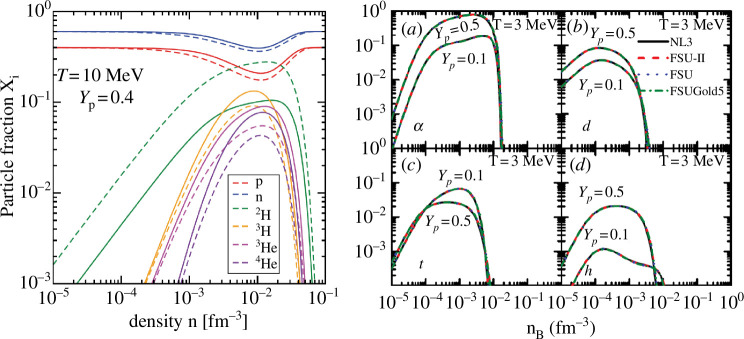
Theoretical predictions of cluster fractions in nuclear matter at finite temperature ( T ) at different proton factions ( $Y_{p}$ ). Left: predictions of the generalized relativistic mean-field model at T=10 MeV and $Y_{p}$ =0.4. Full and dashed lines indicate predictions with and without nucleon–nucleon scattering correlations. Reprinted from [58]. ©2013 with permission from Springer. Right: predictions of the generalized nonlinear relativistic mean-field theory at T=3 MeV and $Y_{p}$ = 0.1, 0.5. Reprinted from [59]. ©2017 with permission from the American Physical Society.

One of the novel features of the project is to probe deuteron, triton, He3 and α clusters on the same footing using the cluster knockout (p,p⁢X) reactions ( X , t , ${\;}^{3}He$ , α ). This benefits from the flexibility of kinematics in the (p,pX) reactions with three-body final states. The flexibility enables one to arbitrarily choose the kinematic condition corresponding to the momentum region of interest for each knocked-out particle, by keeping the quasi-free condition. The interest in probing all of the light clusters is in differences in their spins, isospins and binding energies. In particular, differences in spins may help one to disentangle effects from the tensor and spin–orbit interactions. For example, the AQCM theory predicts that, contrary to the formation of α , a deuteron formation probability is not affected by the spin–orbit interaction because the spins of the proton and the neutron in a deuteron are parallel. The interests in simultaneous studies of clusters are also underpinned by predictions of nuclear matter theories that incorporate clusters. Figure 9 shows theoretical predictions of cluster formation in nuclear matter at finite temperature ( T ) at different proton factions ( $Y_{p}$ ). The generalized relativistic density functional theory at T=10 MeV and $Y_{p}=0.4$ (left panel of figure 9) predicts that the clustering of deuteron, triton and He3 develops, similarly to α , in the region around 1/10 of the saturation density (around 0.01 fm^−3^) with a fraction of ∼ 10% [58]. Relative ratios of cluster formation probabilities determined with the knockout reactions will provide us with a hint to understand the cluster formation mechanisms at the dilute surface of heavy nuclei.

There is also physics specific to each cluster. The formation of a deuteron cluster embodies tensor force effects in nuclei and is closely relevant to p – n short-range correlations (SRC) observed in the electron scattering experiments [60]. The deuteron formation probability in a wide range of nuclei, in particular when compared with the SRC data from the Jefferson laboratory, will provide a more complete picture of the tensor force correlations in nuclei. An interesting aspect of triton and He3 clusters is that their fractions are expected to show distinctive isospin dependences as discussed below.

Changes in the cluster formation probabilities in a long isotope chain spanning stable and unstable isotopes will be a key clue in the revelation of unknown mechanisms. A gradual decrease of the α formation probability with the neutron excess discussed above is considered to reflect the isoscalar (N=Z) nature of the α cluster. If the clustering mechanism is common between deuteron and α , a neutron excess dependence of the deuteron formation probability behaves similarly, while those of triton and He3 clusters would show different trends. Predictions of the generalized nonlinear relativistic mean-field theory [59] shown in the right panel of figure 9 exhibit a clear $Y_{p}$ dependence at T=3 MeV, where the results of $Y_{p}=0.1$ (neutron-rich matter) and 0.5 (symmetric nuclear matter) are compared; triton, a neutron-rich three-nucleon cluster, increases its fraction in neutron-rich matter with $Y_{p}=0.1$ , while the He3 fraction decreases. It is interesting to confirm that similar behaviours can be seen in finite nuclei.

Data for nuclei in a wide mass range are essential in separating several effects: surface-to-volume ratios, Q values and quantum numbers of relevant single-particle orbits. Knockout reaction studies of the α -decay nuclei to compare the (p,p⁢α) cross-sections with α reduced widths can be an interesting approach to α preformation factors [61].

The extensions in cluster species, isospins and masses of nuclei to be investigated benefits from the use of cluster knockout reactions. The proton-induced cluster knockout reactions are natural extensions of (p,2⁢p) and (p,p⁢n) reactions, which have been extensively used in studies of stable and unstable nuclei. Note that [62] concludes by wishing the future success of α knockout reaction studies as ‘An interesting question concerns the existence of quasifree processes involving the knockout of α particles from inner shells of nuclei. The observation of such a process would have a direct bearing on the internal structure of nuclei. This type of experiment may be within the reach of the high-resolution medium-energy accelerators that are just now becoming fully operational.’ In the ONOKORO project, normal-kinematics experiments using high-quality proton beams at 250-400 MeV and double-arm spectrometers (Grand RAIDEN and LAS) available at the RCNP cyclotron facility and inverse-kinematics experiments using stable and unstable heavy-ion beams at HIMAC and RIBF, respectively, are carried out to measure (p,p⁢X) cluster knockout cross-sections.

The accuracy of the cluster formation probabilities derived from the measured (p,p⁢X) cross-sections depends on the reliability of the reaction analyses. DWIA employed in analyses of (p,p⁢N) reactions is a good starting point as well for the cluster knockout reactions and has been applied to (p,p⁢α) reactions [53,63]. For better descriptions of knockout reactions accompanying clusters more weakly bound than an α particle, a theory taking breakup effects into account is being developed. The new theoretical framework called CDCCIA, combining DWIA with the continuum-discretized channel coupling method, has been first applied to (p,p⁢d) reactions [64]. With future improvements, theoretical descriptions of cluster knockout reactions will become as reliable as those of nucleon knockout reactions.

## 6. Summary

Nuclear clusterings are essential in the synthesis of carbon, oxygen and heavy elements and, thus, inevitable in the birth of life. They also play major roles in the dynamics of heavy nuclei, which have close relationships to our society and to future discoveries of new elements. Although well-developed α clusters are known to exist in light N=Z nuclei, and their properties are reasonably well described with modern nuclear structure theories, ‘modestly’ developed clusters in medium to heavy nuclei remain little understood, both theoretically and experimentally. Extension of the focus to include modestly developed clusters leads us to a concept of ‘generalized clusters’ and ‘cluster ubiquitousness’.

The formation of clusters is essentially governed by the competition of two non-central nuclear interactions: the tensor and spin–orbit interactions. The tensor interaction is a key ingredient for the strong binding of the α cluster, whereas the spin–orbit interaction has the effect of breaking clusters and strengthens the symmetry of the shell model. Therefore, the cluster and shell structure compete with each other.

This cluster–shell interplay contributes to the synthesis of light to heavy elements. For instance, the triple- α reaction and C12+α reaction have a cluster structure in the entrance channel, but synthesized nuclei ( C12 and O16 ) after de-excitation have shell-like components. The mixing of both cluster and shell components is essential for the synthesis of many elements.

Clustering determines stability and dynamics, namely, α decays, fission and fusion reactions, of heavy nuclei. Quantitative description of the α preformation factor has been a long-standing problem in nuclear physics. Experimental and theoretical verifications of clusters dynamically produced in the course of fission and fusion reactions will have significant implications for our understanding of heavy-ion dynamics.

Among several experimental techniques for studying clusters, knockout reactions have an advantage in probing clusters in medium to heavy nuclei. The ONOKORO project will provide the most comprehensive data set of cluster knockout reactions for a variety of stable and unstable nuclei spanning a wide mass range up to A=222 . Another distinct feature of the ONOKORO project is to probe all light clusters including d , t , He3 and α . These experimental and theoretical studies are expected to reveal unknown aspects of the cluster formation mechanism and elucidate the phenomena associated with the clusters mentioned above.

## Data Availability

This article has no additional data.

## Footnotes

^1^
Once ^12^C is produced, ^4^He is also produced in the CNO cycle.
